# High glucose induces apoptosis and suppresses proliferation of adult rat neural stem cells following *in vitro* ischemia

**DOI:** 10.1186/1471-2202-14-24

**Published:** 2013-03-04

**Authors:** Jian Chen, Yang Guo, Wei Cheng, Ruiqing Chen, Tianzhu Liu, Zhenzhou Chen, Sheng Tan

**Affiliations:** 1 Key Laboratory of Brain Function Repair and Regeneration of Guangdong, Department of Neurology, Zhujiang Hospital, Southern Medical University, Guangzhou, China; 2 Department of Neurosurgery, Zhujiang Hospital, Southern Medical University, Guangzhou, China

**Keywords:** Neural stem cells, Hyperglycemia, Proliferation, Apoptosis, Mitogen-activated protein kinases (MAPKs)

## Abstract

**Background:**

Post-stroke hyperglycemia appears to be associated with poor outcome from stroke, greater mortality, and reduced functional recovery. Focal cerebral ischemia data support that neural stem cells (NSCs) play an important role in post-ischemic repair. Here we sought to evaluate the negative effects of hyperglycemia on the cellular biology of NSCs following anoxia, and to test whether high glucose affects NSC recovery from ischemic injury.

**Results:**

In this study, we used immortalized adult neural stem cells lines and we induced *in vitro* ischemia by 6 h oxygen and glucose deprivation (OGD) in an anaerobic incubator. Reperfusion was performed by returning cells to normoxic conditions and the cells were then incubated in experimental medium with various concentrations of glucose (17.5, 27.75, 41.75, and 83.75 mM) for 24 h. We found that high glucose (≥27.75 mM) exposure induced apoptosis of NSCs in a dose-dependent manner after exposure to OGD, using an Annexin V/PI apoptosis detection kit. The cell viability and proliferative activity of NSCs following OGD *in vitro*, evaluated with both a Cell Counting kit-8 (CCK-8) assay and a 5-ethynyl-2’-deoxyuridine (EdU) incorporation assay, were inhibited by high glucose exposure. Cell cycle analysis showed that high glucose exposure increased the percentage of cells in G0/G1-phase, and reduced the percentage of cells in S-phase. Furthermore, high glucose exposure was found to significantly induce the activation of c-Jun N-terminal protein kinase (JNK) and p38 mitogen-activated protein kinase (MAPK) and suppress extracellular signal-regulated kinase 1/2 (ERK1/2) activity.

**Conclusions:**

Our results demonstrate that high glucose induces apoptosis and inhibits proliferation of NSCs following OGD *in vitro*, which may be associated with the activation of JNK/p38 MAPK pathways and the delay of G1-S transition in the cells.

## Background

Stroke has become the leading cause of morbidity and mortality in China, amounting to 1.65 million deaths and 2 million new onsets every year, rising on average by 8.4% each year [1]. Despite intensive investigations into the mechanism and treatment of stroke, very limited effective therapies are available for stroke patients. Compared with the very limited traditional therapies, such as neuroprotective strategies, some new neuroregenerative therapies involving endogenous or exogenous approaches are promising. Recent reports have shown that endogenous and transplanted neural stem cells (NSCs) can be activated by cerebral ischemia and take part in the regeneration of neural function [2,3]. However, some basic questions concerning the fate of NSCs after an ischemic or hypoxic insult remain to be answered. Low survival and insufficient neuronal differentiation of endogenous or engrafted NSCs within the ischemic core and peri-infarct regions hamper the efficacy of NSC therapy and limit its clinical applications [4]. Therefore, understanding the cellular biology of NSCs within an ischemic or hypoxic environment could give rise to new possibilities for controlling the fate of NSCs, leading to the development of novel cell replacement therapies after ischemic stroke.

NSCs within the adult brain germinal centers reside in a specialized micro-environmental niche that regulates cell migration, adhesion, proliferation and differentiation under both physiological and pathological conditions [5-7]. Among various incidents during cerebral ischemia, the reduction in the supply of oxygen (hypoxia) and glucose (hypoglycemia) in the brain is a major factor mediating neural damage [8]. It has recently been reported that the proliferation of cultured NSCs is promoted by hypoxia, and NSCs are resistant to ischemia-induced apoptosis [9,10]. However, there are a low number of NSCs that can survive for a long time and contribute to the reconstruction of neural circuitry [4]. It is believed that the glucose level is a crucial factor for the self-renewal and multipoietic activities of NSCs following cerebral ischemia. There are reports showing that low glucose suppresses the proliferation and increases the differentiation of cultured NSCs *in vitro*[8], and that post-stroke hyperglycemia is seen in up to 50% of patients who have an initial blood glucose above 6.0–7.0 mM [11,12]. Hyperglycemia appears to be associated with more severe stroke, assessed either with a clinical stroke scale [13] or by lesion volume. Plasma glucose is an important determinant of brain injury in experimental models of focal cerebral ischemia/reperfusion [14], but few studies have explored the effect of high glucose on NSCs or progenitor cells following oxygen and glucose deprivation/reperfusion (OGD/R) insult.

In this study, we first assessed the effects of high glucose on the proliferation and apoptosis of NSCs using an *in vitro* ischemic model. We further examined whether the activation of mitogen-activated protein kinase (MAPK) signaling molecules is involved in the proliferation and apoptosis of NSCs, as MAPK signaling plays an important role in central nervous system (CNS) development and differentiation [15]. We found that mild elevated glucose facilitated the survival of NSCs after hypoxia, whereas higher glucose exacerbated the hypoxia-mediated injury, with a G1/S transition delay and the activation of c-Jun N-terminal kinase (JNK) and p38 MAPK signaling molecules. These findings may have important implications for glycemic control in stroke patients, and provide a further understanding of the fate of NSCs following cerebral ischemia.

## Methods

### Cell culture

Adult rat neural stem cells (NSCs) were purchased from Chemicon, Inc.(Billerica, MA, USA), and maintained by an adherent monoculture method developed by Palmer et al. [16]. It is recommended to grow the cells in the routine commercial Neural Stem Cell Basal Media (Cat. No. SCM009, Millipore, Billerica, MA, USA) containing 17.5 mM glucose (used as the control level in this study), which has been optimized for the growth and *in vitro* differentiation of NSCs derived from rodents. To estimate the effects of high glucose on the survival and proliferation of NSCs following *in vitro* ischemia, we used *in vitro* concentrations of 27.75, 41.75, and 83.75 mM glucose, which are similar to *in vivo* levels of glucose under "diabetes mellitus", "diabetic ketoacidosis", and "hyperglycemia hyperosmolar status" conditions, respectively. High glucose conditions (27.75, 41.75, and 83.75 mM) were established by addition of D-glucose to Neural Stem Cell Basal Media. Briefly, the cells were plated onto poly-L-ornithine- and laminin- (Cat. No. P3655, L2020, Sigma-Aldrich, Inc., St. Louis, MO, USA) coated 60 mm culture dishes or 96-well plates. After reaching 50% confluence, the cells were left in anoxic conditions for an appropriate duration to induce an OGD/R insult. The cells were then exposed to the experimental media with various concentrations of D-glucose for 24 h. Mannitol was used as a control to exclude a possible effect of osmolality on cell viability. We changed the mannose concentrations to keep the osmotic pressure of the culture medium at various glucose concentrations. Thereafter, cells were harvested for analysis.

### Oxygen glucose deprivation/reperfusion procedure

To induce OGD, NSCs were grown in 60 mm culture dishes or 96-well plates for 24 h. Then they were washed twice with Earle’s balanced salt solution (EBSS) (g/L: NaCl 6.80, KCl 0.4, CaCl_2_ 0.2, MgSO_4_ 0.2, NaH_2_PO_4_ 1.14, NaHCO_3_ 2.2, phenol red 0.02). The cells were then immersed in 5 mL (for 60 mm petri dishes) or 100 μL (for 96-well plates) of glucose-free NBM-B27 media (Neurobasal glucose-free, Invitrogen, Carlsbad, CA, USA) with 25 mM L-glutamate (Sigma–Aldrich) before the plates were transferred into a CO_2_/O_2_ tri-gas incubator (Forma 3131, Thermo Fisher Scientific Inc., Asheville, NC, USA) with an atmosphere of 1% O_2_, 5% CO_2_ and 94% N_2_, 98% humidity at 37°C. The incubator was flooded with pre-warmed and humidified gas consisting of 5% (v/v) CO_2_ in 95% N_2_. Oxygen and CO_2_ content in the wells were continuously maintained at a constant level by the tri-gas incubator with a precise gas sensor. The cells were left in the incubator for different durations (2, 4, 6, 8 and 10 h). Reperfusion was performed by removing the plates from the incubator, immediately washing twice with EBSS and adding an equal volume of neural stem cell basal medium supplemented with 20 ng/mL basic fibroblast growth factor (b-FGF) (Millipore, Cat. No. GF003). The cells were then returned to a CO_2_ incubator (Forma 3110, Thermo Fisher Scientific Inc.) with an atmosphere of 5% CO_2_, 95% air, and 98% humidity at 37°C for 24 h. To induce OGD/R of NSCs *in vitro*, the induction of 50% apoptosis in NSCs was considered appropriate. Cells were examined by light microscopy (IX700, Olympus, Tokyo, Japan) for qualitative assessment of NSC damage. For quantitative measurements of cell viability, we used a WST-8 assay (Dojindo Laboratories, Kumamoto, Japan).

### Cell viability tests

To estimate the number of viable cells, approximately 50,000 cells were grown in each well of poly-L-lysine-coated 96-well plates with 100 μL medium. We performed a WST-8 assay with the Cell Counting Kit-SF (Dojindo Laboratories, Kumamoto, Japan) using the methods described by Horie et al [8]. Cell Counting Kit-8 solution was added to the cell culture medium to a final concentration of 5 μL/100 μL, and incubated for an additional 4 h at 37°C. We measured the absorbance at 450 nm with a reference wavelength of 630 nm with a microplate reader (ELx800, BioTek instruments, Inc., Winooski, VT, USA). In each experiment, at least three parallel wells were set up. Using these experimental procedures, we obtained a good linear relationship between the net absorbance and the viable cell density.

### EdU incorporation assay

We assessed proliferation of the cells using the 5-ethynyl-2’-deoxyuridine (EdU) incorporation assay. NSCs were incubated with EdU to see which fraction of cells showed proliferative activity. The EdU incorporation assay was performed with a Cell-Light EdU kit (Ribobio Co., Ltd., Guangzhou, China) according to the manufacturer’s instructions. Briefly, NSCs were cultured in a well of a 96-well plate coated with poly-D-lysine at a cell density of 5000 cells per well, and the cells were then labeled with 50 μM EdU (1:1000) and incubated for an additional 2 h before the cells were fixed with 4% formaldehyde for 15 min at room temperature and treated with 0.5% Triton X-100 for 20 min at room temperature for permeabilization. After washing with PBS three times, each well of cells was reacted with 100 μL of 1× Apollo® reaction cocktail for 30 min. Subsequently, the DNA content of each well of cells was stained with 50 μL DAPI (Vector Laboratories, Inc., Burlingame, CA, USA) for 30 min and mounted. EdU-labeled cells were counted using fluorescence microscopy (CKX41-F32FL, Olympus). and normalized to the total number of DAPI-stained cells.

### Cell cycle analysis

The effect of different concentrations of glucose on the cell cycle was measured by flow cytometry, as described by Chen et al. [17]. Briefly, NSCs at 1 × 10^6^ cells per plate were cultured in 60 mm plates coated with poly-D-lysine. At the end of the experiments, cells were dissociated using Accutase™ (Cat. No. SCR005, Millipore) and harvested, followed by 75% ice cold ethanol fixation overnight at -20°C. Fixed cells were stained with propidium iodide (BD Biosciences, San Jose, CA, USA) (50 μg/mL) containing 50 μg/mL RNase A (BD Biosciences) for 30 min at 37°C in the dark, and subsequently analyzed by fluorescence-activated sorting (FACSCalibur, BD Biosciences). We evaluated the changes in cell cycle distribution and calculated the proliferation index (PI) and S-phase cell fraction (SPF). The following formula was used: PI = (S + G2/M)/(G0/G1 + S + G2/M), SPF = S/(G0/G1 + S + G2/M).

### Assessment of necrosis and apoptosis of NSCs

The quantitative assessment of NSC necrosis and apoptosis was performed by flow cytometry. Briefly, NSCs were cultured in 60 mm plates coated with poly-D-lysine at a density of 1 × 10^6^ cells per plate. At the end of the experiments, cells were dissociated using Accutase™ and harvested, then stained with Annexin V and PI for 15 min at 37°C in the dark using the Annexin V-FITC apoptosis detection kit (BD Biosciences). Subsequently, the labeled cells were assessed by a FACSCalibur instrument.

### Immunostaining

To confirm neural stem and/or progenitor status of the starting cell population, immunostaining was performed using the protocol below. NSCs seeded in a 96-well plate were fixed in PBS containing 4% paraformaldehyde for 30 min at room temperature and permeabilized by incubation with 0.3% Triton X-100 for 20 min. After three washes with PBS, the cells were blocked with 10% normal goat serum (Invitrogen) for 30 min. The cells were then incubated overnight with anti-nestin antibody (Cat. No. sc-58813, 1:800, mouse monoclonal antibody, Santa Cruz Biotechnology, Inc., Santa Cruz, CA, USA). After removal of the primary antibody solution, the cells were washed with PBS three times and incubated with secondary antibody (Alexa Fluor® 594 goat anti-mouse IgG, Cat. No. 115-585-003, The Jackson Laboratory, Sacramento, CA, USA) and 5 μg/mL of DAPI for nuclear staining for 4 h at 37°C under light-shading conditions. After three washes, the cells were mounted with Perma-Fluor Aqueous Mounting Medium (Thermo Fisher Scientific Inc.) and the fluorescent images were viewed and captured under a fluorescence microscope (Olympus). For estimation of the homogeneity of NSCs, we counted the numbers of nestin-positive cells (positive NSCs) during different passages.

### Western blot analysis

To determine the amounts of phosphorylated ERK, JNK and p38, cells were washed with PBS and harvested with RIPA lysis buffer (20 mM Tris-HCl, pH 7.6, 150 mM NaCl, 1% Triton X-100, 2 mM PMSF, KeyGen Biotech, Nanjing, China) containing a protease and phosphatase inhibitor cocktail (KeyGen Biotech), and incubated on ice for 30 min. All cell lysates were cleared by centrifugation (14,000 × g for 20 min at 4°C). Protein concentrations were quantified by BCA assay (KeyGen Biotech), and equal amounts of protein from each sample were boiled for 5 min in sample buffer containing 62.5 mM Tris-HCl, pH 6.8, 2% SDS, 5% glycerol, 2.5% β-mercaptoethanol, and 0.1% bromophenol blue (KeyGen Biotech). Protein samples were fractionated by 10% SDS-polyacrylamide gel electrophoresis in electrophoresis buffer containing 25 mM Tris-HCl, 192 mM glycine, and 0.1% SDS for 30 min at 80 V and 90 min at 120 V, and transferred onto a polyvinylidene difluoride membrane (EMD Millipore, Darmstadt, Germany) for 1 h at 80 V. The membranes were blocked with a blocking buffer containing 20 mM Tris-HCl, pH 7.6, 137 mM NaCl, and 0.1% Tween 20 (TBST) supplemented with 5% non-fat milk overnight at 4°C. The following primary antibodies were used and incubated for 4 h at 4°C: rabbit monoclonal anti-p-ERK 1/2 (Cat. No. 3179S, 1:500, Cell Signaling Technology, Inc., Danvers, MA, USA), mouse polyclonal anti-ERK1/2 (Cat. No. sc-135900, 1:150, Santa Cruz Biotechnology, Inc.), mouse monoclonal anti-p-JNK (Cat. No. 9255S, 1:500, Cell Signaling Technology, Inc.), rabbit polyclonal anti-JNK2 (Cat. No. sc-827, 1:150, Santa Cruz Biotechnology, Inc.), rabbit polyclonal anti-p-p38 (Cat. No. 9216, 1:500, Cell Signaling Technology, Inc.), rabbit polyclonal anti-p38 (Cat. No. sc-7149, 1:150, Santa Cruz Biotechnology, Inc.). Subsequently, the membrane was incubated with a secondary antibody conjugated with IRDye® infrared dyes (LI-COR Biosciences, Lincoln, NE, USA).at a 1:15,000 dilution in TBST for 1 h. Signals were detected by an Odyssey® CLx Infrared Imaging System (LI-COR Biosciences). The housekeeping protein β-actin was used as a control and tested simultaneously with a mouse monoclonal antibody (Cat. No. sc-47778, 1:500, Santa Cruz Biotechnology, Inc.). Western blotting data were analyzed with Gel-Pro analyzer software 4.0 (Media Cybernetics, Rockville, MD, USA), and the ratios of phosphorylated ERK2/total ERK2, phosphorylated JNK/total JNK and phosphorylated p38/total p38 pixels were calculated.

### Statistics

All results were collected as the average of at least six independent experiments. Data are presented as the mean ± standard deviation (SD). SPSS version 13.0 (SPSS, Chicago, IL, USA) was used for statistical analysis. Statistical analysis of the data for multiple comparisons was performed by one-way analysis of variance, and the Bonferroni test was used for post hoc comparison to controls. A value of P < 0.05 was considered statistically significant.

## Results

### A simple, stable and reliable model of NSC OGD/R was successfully established *in vitro*

**Figure 1:**
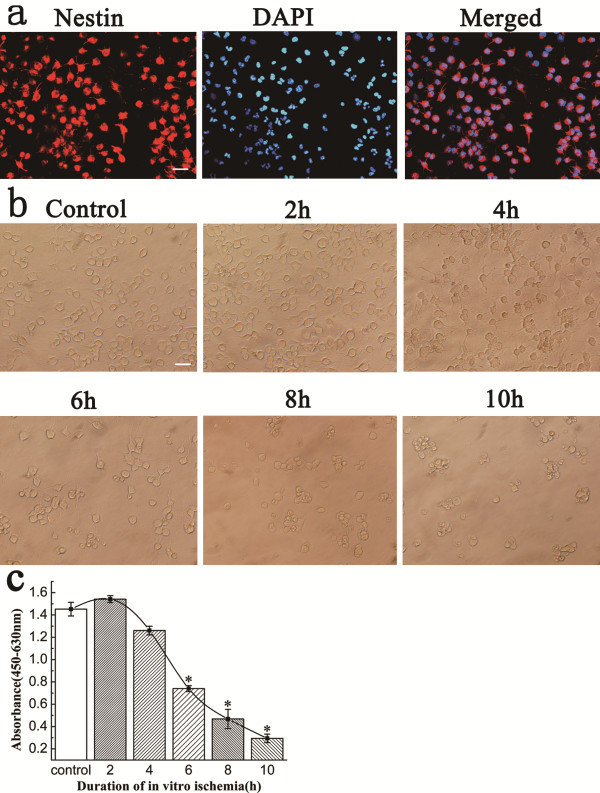
**Establishment of an adult neural stem cell *in vitro *model of ischemia.** (**a**) The identification of adult neural stem cells throughout different passages. Immunocytochemical detection of nestin (red) was performed. The nuclei of NSCs were revealed by DAPI staining (blue). The scale bar represents 20 μm. (**b**) Digital photomicrographs of NSCs exposed to different durations of *in vitro* ischemia. NSCs were subjected to OGD for different periods (0–10 h), then were returned to normoxic conditions and incubated for an additional 24 h. The cells were photographed at the end of the experimental period. All photomicrographs are from different sister cultures from the same plating. Minor adjustments to brightness, contrast and color balance have been made to the digital images. The scale bar represents 20 μm. (**c**) Cell viability and survival rate of the NSCs following *in vitro* ischemia. To estimate the number of viable cells, approximately 50,000 cells were grown in each well of poly-L-lysine-coated 96-well plates with 100 μL medium and the absorbance at 490 nm was directly proportional to the number of viable NSCs per well at each time point. Data points represent the mean ± SD of six independent experiments. *P < 0.05 versus control, analyzed by one-way ANOVA/Bonferroni post hoc test.

To confirm the neural stem and/or progenitor status of the starting cell population, NSCs were subjected to immunocytochemistry before the anaerobic incubation. We found that the great majority (94.6%) of cells expressed nestin, a neural progenitor marker (Figure 1a), which indicates that most of the cells had stem and/or progenitor status. OGD/R was induced by a wash in glucose-free EBSS prior to a 2–10 h anaerobic incubation followed by a 24 h post-incubation period. In this model (Figure 1b), *in vitro* ischemia ≤ 2 h resulted in little or no injury of NSCs, while ischemia between 4–6 h produced mild to moderate injury, characterized by cell shrinkage with few or no cells swelling. Ischemia > 6 h caused progressive NSC apoptosis and the percentage of apoptotic cells increased to 50–90%. The results of the Cell Counting kit (CCK)-8 assay for viability reflected the light-microscopic observations of cell death. Ischemic incubation > 6 h decreased the cell survival rates to 50% (Figure 1c). We found that 6 h of ischemic incubation was a threshold, as cell survival rates decreased dramatically in response to *in vitro* ischemia after this time point. Thus, an *in vitro* ischemia incubation time > 6 h is necessary to induce significant cell injury.

### High glucose diminished the proliferation of NSCs following *in vitro* ischemia

**Figure 2:**
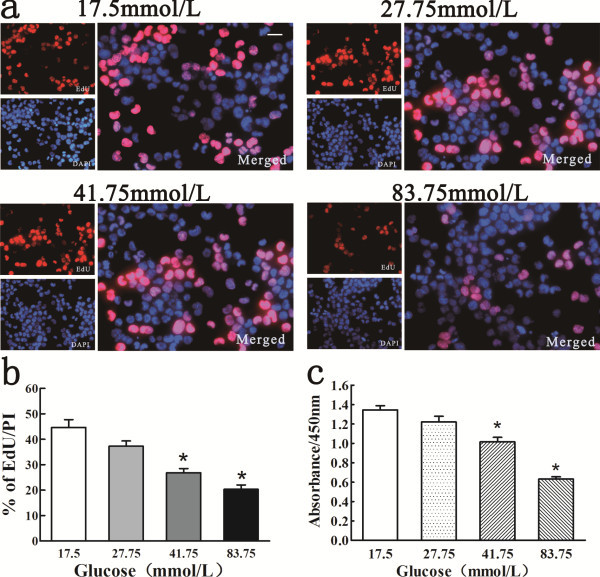
**The proliferation and viability of NSCs in high glucose were analyzed using the EdU incorporation assay and CCK-8 assay.** (**a**) EdU-labeled cells appear in purple as the EdU (red) is colocalized with DAPI (blue). The scale bar represents 20 μm. (**b**) Note the decreased number of EdU-positive cells in NSCs exposed to high glucose (41.75 or 83.75 mM), indicating less proliferation. However, there was no difference in EdU incorporation between the 27.75 mM glucose treatment and control. % of EdU/DAPI means percentage of EdU-positive cells in NSCs. *P < 0.01 versus control, analyzed by one-way ANOVA/Bonferroni post hoc test versus the normal glucose group (17.5 mM glucose). (**c**) High glucose exposure caused a significant decrease in the viability of NSCs following OGD. *P < 0.01 versus control, analyzed by one-way ANOVA/Bonferroni post hoc test.

We evaluated the proliferative activity of NSCs incubated in various concentrations of glucose for 24 h after 6 h ischemia. 5-ethynyl-2’-deoxyuridine (EdU) incorporation was decreased compared with 17.5 mM glucose (control) when cell cultures were exposed to higher glucose (Figure 2a, 2b). We found that cell viability for all the cultures examined was over 90% at the beginning of the experiment. As shown in Figure 2c, the viable cells in 27.75 mM glucose medium were approximately 90.73% ± 10.63% of control, and the viable cells in the higher glucose concentrations, 41.75 and 83.75 mM, were reduced to 75.46% ± 8.53% and 46.92% ± 4.34% of control, respectively, suggesting that the proliferation of NSCs was further suppressed by higher glucose concentrations. Compared with the control, there was a significant decrease in EdU incorporation and cell viability in the cultures exposed to 41.75 and 83.75 mM glucose (P < 0.05), but not in the cells exposed to 27.75 mM glucose. The influence of osmolality on cell viability was excluded in this study (Table 1). These findings suggest that continuous culture at a moderately high concentration of glucose (27.75 mM) did not significantly inhibit the proliferative activity of NSCs, but higher concentrations of glucose (41.75 or 83.75 mM) significantly diminished the proliferation potential of NSCs.

**Table 1:** The effect of osmolality on the viability of adult NSCs (n = 6, mean ± SD)

**Group**	**D-glucose**	**D-glucose + D-Mannitol**	**P**
Control	2.346 ± 0.033	—	—
27.75 mM	2.283 ± 0.044^*^	2.257 ± 0.044	0.324
41.75 mM	2.165 ± 0.041^*^	2.201 ± 0.068	0.293
83.75 mM	1.330 ± 0.029^*^	1.319 ± 0.574	0.679
F	955.900	502.976	
*P*	0.000	0.000	

*P < 0.05 vs. control.

### High glucose decreased the proliferation of NSCs following *in vitro* ischemia by delaying the G1-S transition

**Figure 3:**
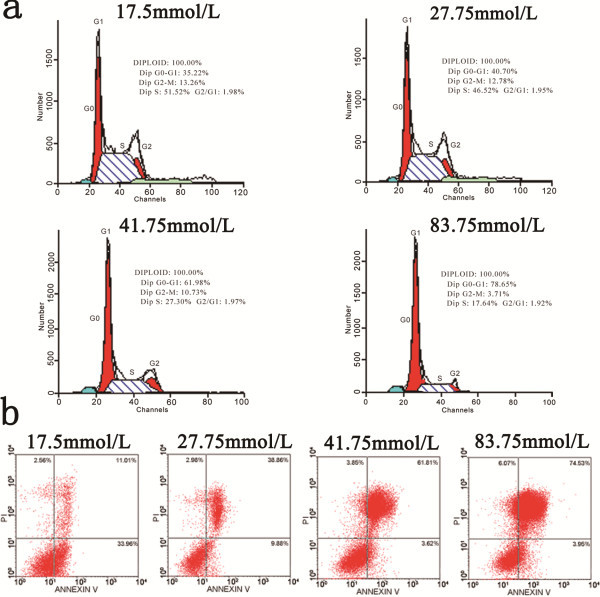
**The effect of various concentrations of glucose on the cell cycle and apoptosis of NSCs following *in vitro *ischemia.** (**a**) The cell cycle distribution of NSCs was analyzed by flow cytometry. The percentage of NSCs in G0/G1-phase was significantly increased, whereas the percentage of NSCs in S-phase was markedly decreased, compared with the control in all there higher glucose groups. (**b**) The apoptosis of NSCs was quantified by flow cytometry. Relative fluorescence in NSC populations was double-stained with Annexin V (AV)-FITC and propidium iodide (PI). In each panel, AV-/PI- cells are viable NSCs, shown in the lower-left quadrant. The AV+/PI + cells represent late apoptotic NSCs, shown in the upper-right quadrant, and the AV+/PI- cells represent early apoptotic NSCs, shown in the lower-right quadrant. AV-/PI + cells, representing necrotic NSCs, are shown in the upper-left quadrant. The proportion of apoptotic cells (both in early and late phase apoptosis) is higher in all the other three glucose groups compared with the control.

We analyzed the cell cycle of NSCs by fluorescence-activated cell sorting (FACS) after treatment with different concentrations of glucose. As illustrated in Table 2, we identified approximately 66.5% of NSCs in mitotic phase in the total NSCs cultured in the basal culture medium control (17.5 mM glucose) (proliferation index (PI) = 0.665 ± 0.142). PI and S-phase cell fraction (SPF) were significantly decreased compared with the control in the cultures exposed to higher glucose (P < 0.01). The percentage of cells in G0/G1-phase increased significantly, whereas the percentage of cells in S-phase decreased compared with the control in all the higher glucose groups (Figure 3a).

**Table 2:** **Effect of glucose on the cell cycle of NSCs following *in vitro *ischemia (n = 6, mean ± SD)**

**Glucose (mM)**	**PI**	**P**	**SPF**	**P**
17.5	0.665 ± 0.142	-	0.515 ± 0.014	-
27.75	0.595 ± 0.026	0.002	0.465 ± 0.042	0.016
41.75	0.382 ± 0.019	0.000	0.273 ± 0.024	0.000
83.75	0.217 ± 0.042	0.000	0.176 ± 0.008	0.000
F	333.252		239.024	
P	0.000		0.000	

Proliferation Index (PI) = (S + G2/M)/(G0/G1+ S + G2/M), S-phase cell Fraction (SPF) = S/(G0/G1 + S + G2/M). The data are expressed as the mean ± SD of six independent experiments, and analyzed by one-way ANOVA followed by Bonferroni test versus the normal glucose group (17.5 mM glucose).

### High glucose induced apoptosis of NSCs following *in vitro* ischemia

To determine the effects of various concentrations of glucose on NSC apoptosis, we performed flow cytometry to analyze glucose-mediated apoptosis of NSCs after OGD. After reoxygenation in 17.5, 27.75, 41.75, and 83.75 mM glucose medium for 24 h after 6 h of hypoxic/ischemic treatment, the percentages of apoptotic NSCs were 11.01 ± 0.61%, 38.86 ± 4.94%, 61.81 ± 3.53%, and 74.53 ± 0.77%, respectively. The percentage of apoptotic cells was significantly increased in the three higher glucose groups (27.75, 41.75, and 83.75 mM glucose) compared with the control group (17.5 mM glucose) (P < 0.01) (Figure 3b).

### High glucose activated JNK and p38 MAPKs in NSCs following *in vitro* ischemia

**Figure 4:**
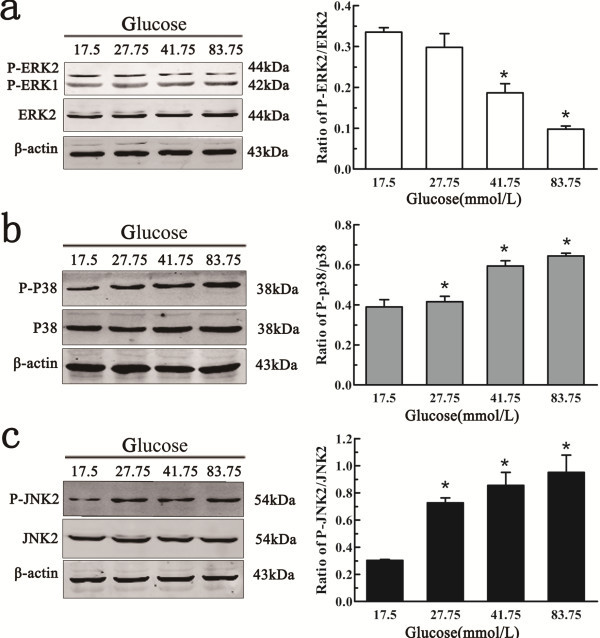
**The effect of various concentrations of glucose on the activation of ERK, JNK and p38 MAPKs in NSCs following *in vitro *ischemia.** Significant changes in the levels of phosphorylated ERK, JNK and p38 were observed in NSCs exposed to different concentrations of glucose following *in vitro* ischemia. (**a**) The p-ERK2 level was decreased in NSCs treated with 41.75 and 83.75 mM glucose after 6 h ischemic exposure, but no significant change in the p-ERK2 level was observed between the normal glucose group (17.5 mM) and the 27.75 mM glucose group. (**b-c**) Both p-p38 and p-JNK significantly increased in NSCs of the three glucose treatment groups (27.75, 41.75, and 83.75 mM) compared with the normal glucose group cells. *P < 0.05 versus control, analyzed by one-way ANOVA/Bonferroni post hoc test.

To further understand the mechanism by which high glucose suppressed the proliferation of NSCs, we investigated the phosphorylation of ERK, JNK and p38 MAPK in NSC cultures treated with different concentrations of glucose. As shown in Figure 4, total ERK2, total JNK2 and total p38 were consistently expressed and no significant changes were found between the control group and the higher glucose groups. For the phosphorylated proteins, we found that the level of p-ERK significantly decreased in the NSCs treated with 41.75 and 83.75 mM glucose after 6 h of ischemia, but there were no significant changes in the cells treated with 27.75 mM glucose compared with the control (Figure 4a). The levels of p-p38 and p-JNK2 significantly increased in the three higher glucose groups (27.75, 41.75, and 83.75 mM glucose) compared with the control (Figure 4b, 4c).

## Discussion

Recent studies have demonstrated the potential for endogenous and transplanted neural stem/progenitor cells (NSPCs) to ameliorate the structural and behavioral deficits associated with cerebral ischemia in animal models [2], providing a potential therapy for ischemic stroke. However, poor NSPC survival within the ischemic core and peri-infarct regions following stroke has hampered the benefits and applications of cell-based therapies [18,19]. Many factors are involved in the regulation of the biological behaviors of NSCs, including genetics, growth factors, neurotransmitters, stress, hormones, and environmental factors like hypoxia. Recent studies have shown that the availability of glucose, but not of oxygen, is a restricting factor for NSC survival and proliferation following hypoxic/ischemic damage [9]. Furthermore, the proliferation of certain developmental stage-specific cells, such as embryonic and postnatal NSCs, has been proven to be dependent on the glucose concentration under physiological and pathological conditions such as diabetes [20-22]. It is increasingly evident that post-stroke hyperglycemia is associated with poor outcome, and seems to particularly affect outcome in patients without diabetes [23,24]. With regard to cerebral ischemia/reperfusion pathophysiology, it is reported that hyperglycemia exacerbates brain injury due to poor blood flow to the ischemic penumbra, accumulation of lactate and intracellular acidosis in the ischemic brain [25-27], and enhancement of the inflammatory response [26]. Whether the harmful effects of hyperglycemia are mediated by exacerbating the ischemic injury in NSCs or NPCs is unclear. So far, little is known about the effect of high glucose on the proliferation of adult neural stem cells following *in vitro* ischemia. In this study, we found that exposure to high glucose induced apoptosis of NSCs in a dose-dependent manner and inhibited the viability and proliferation of NSCs following OGD *in vitro*. Furthermore, we observed prolonged activation of JNK/p38 MAPK, suppressed ERK1/2 activity, and an increased percentage of cells in G0/G1-phase in NSCs treated with high glucose. In conclusion, our results indicate that high glucose induces the apoptosis and inhibits the proliferation of NSCs following OGD *in vitro*, which may be associated with a prolonged activation of JNK/p38 MAPK pathways and a delay of the cell G1-S transition.

Since glucose concentrations can be controlled and the actions of extrinsic factors can be delineated in an *in vitro* culture system, we used immortalized adult NSCs to investigate the effects of high glucose on the proliferation of NSCs using a well-characterized *in vitro* OGD model. The NSCs were isolated from the hippocampus of adult Fisher 344 rats, widely used for a variety of research applications including drug development, studies of neurotoxicity, neurogenesis, electrophysiology, neurotransmitter and receptor functions, and CNS disorders. In NSC cultures, the majority of cells kept their neural stem and/or progenitor status during the different passages. Most *in vitro* models of ischemia using neuronal cultures have used OGD to mimic the reduced intracellular energy state that occurs in neuronal cells following permanent and transient cerebral ischemia [28-30]. These models have been used to assess whether agents exacerbate or reduce *in vitro* neuronal ischemic injury [31,32]. However, the duration of OGD that was required to induce NSC ischemic injury was reported to vary in different *in vitro* models of ischemia. Additionally, it is common practice to culture cells in a sealed hypoxia chamber to mimic anoxic conditions, in which the O_2_ level is usually approximately 0% [33], while the O_2_ level observed in the anoxic environment often remains unchanged between 0% and 1%. In our study, we used a tri-gas incubator to adjust the O_2_ level in the cultures to a constant level (1%), and determined appropriate anaerobic incubation times by modifying and incorporating features used in neuronal *in vitro* models of ischemia [34]. We thus determined that 6 h of OGD incubation mimicked cerebral hypoxic-ischemic injury.

*In vitro* systems used to study neuronal responses to changes in ambient glucose concentrations must consider that the glucose levels *in vitro* should be of practical relevance to the brain *in vivo*[35,36]. The physiological or normal blood glucose concentration *in vivo* can range from 5.5–7.0 mM. Thus, 5.5 mM is usually recognized as “englycemic” *in vitro* culture conditions for CNS research. However, it is not applied to the *in vitro* NSC culture media that are usually used (e.g. DMEM/F12), which normally contain 17.5 mM glucose, a level perhaps seen in the plasma of obese ob/ob mice. At the beginning of our study, we were puzzled as to why the NSCs must be grown in media with such a high glucose concentration, rather than in media with lower glucose concentrations, such as 7.0 mM and 5.5 mM. To address this question, we conducted initial experiments using 7.0 mM and 5.5 mM glucose to mimic diabetic and physiological glucose levels *in vivo*, respectively. The viability of NSCs exposed to 5.5, 7.0, and 17.5 mM glucose medium for 24, 48 or 72 h was examined by MTS assay (Figure S1, see Additional file 1, available online). The relative increase in the number of NSCs in each group was represented by the ratio of 72 h viability to 24 h viability (Figure S2, see Additional file 1). We found that the NSCs could not be grown in the medium with 5.5 or 7.0 mM glucose, but grew well in the medium with 17.5 mM glucose. To evaluate the effects of high glucose on the survival and proliferation of NSCs following *in vitro* ischemia, 17.5 mM glucose was chosen as the control, and higher concentrations of glucose were contained in the experimental medium. Thus, we used *in vitro* concentrations of 27.75, 41.75, and 83.75 mM glucose, which are similar to *in vivo* levels of glucose under “diabetes mellitus”, “diabetic ketoacidosis”, and “hyperglycemia hyperosmolar status” conditions, respectively.

High glucose concentrations are known to have detrimental effects on many cell types, by impairing cellular functions and inducing cell apoptosis. High glucose has been shown to inhibit the proliferation, migration and *in vitro* angiogenic capacity of bone marrow-derived endothelial progenitor cells [37] and to alter the regenerative potential of mesenchymal stem cells [38]. Furthermore, hyperglycemic conditions affect the proliferation and apoptosis of NPCs in the developing spinal neural tube, leading to abnormal development [39]. If elevated levels of glucose are detrimental to neuronal survival during ischemia, does high glucose (up to 40 mM) damage neurons and NPCs? In the present study, exposure to high glucose (up to 41.75 mM for 24 h) decreased viability and proliferation and increased apoptosis in NSCs following *in vitro* ischemia. Our results are consistent with the studies reported above, but we used different concentrations of glucose. Meanwhile, our study also showed that high glucose treatment consistently suppressed DNA duplication and cell division of NSCs following *in vitro* ischemia by blocking the G1-S transition of the cell cycle.

We further examined the regulatory effect of high glucose on the activation of signaling molecules from the MAPK pathways. MAPKs include three major families: extracellular signal-regulated kinases 1/2 (ERK 1/2), c-Jun N-terminal kinases (JNK), and p38 MAPKs (p38). Upon their activation by the phosphorylation of Thr and Tyr residues, MAPKs regulate cellular processes such as proliferation, survival/apoptosis, differentiation, development, adherence, motility, metabolism, and gene regulation [40]. In the central nervous system, MAPKs are relatively highly expressed. Previous studies suggested that the expression or phosphorylation levels of MAPKs drastically changed in post-ischemic brain tissues, and that the inhibition of MAPK cascades could alter the outcome of ischemic brain injury in *in vitro* and *in vivo* experimental models [41,42]. Therefore, we examined the phosphorylation levels of ERK1/2, JNK and p38 in NSCs exposed to different concentrations of glucose after OGD. We found that the level of p-ERK2 decreased, while the levels of p-p38 and p-JNK2 increased in the cells treated with the three higher glucose concentrations (27.75, 41.75, and 83.75 mM glucose) compared with the control. It has been reported that the role of ERK1/2 in ischemia-mediated neuronal death is disputable [43]. Despite the volume of evidence supporting that the elevation of p-ERK1/2 after ischemic injury is a detrimental effect essential for oxidative stress and inflammation-related cell death, numerous studies have demonstrated that ERK1/2 activation contributes to the protective effects of many neuroprotectants. Our results showed that high glucose decreased ERK2 phosphorylation in OGD NSCs, resulting in less proliferation. Because JNK2 and p38 are generally activated by the same stress signals, such as osmotic shock and heart shock, they are referred to as stress-activated protein kinases (SAPKs). Phosphorylation of the p38 pathway can induce cell apoptosis and inhibition of p38 with SB203580 can reduce cell death [44,45]. In addition, JNK also stimulates cell apoptosis and inhibits cell proliferation when it is activated by cell stress [46]. The increased levels of p-JNK2/p-p38 and the decreased level of p-ERK2 observed in our experiments may reflect a new balance between cell growth and cell death after cells are exposed to high glucose treatment following *in vitro* ischemia.

## Conclusions

Taken together, our data suggest that mild elevated glucose after hypoxia may improve NSC recovery from ischemic injury, while higher glucose may exacerbate the ischemic injury through activation of JNK and p38 MAPK signaling pathways. The actual mechanism by which high glucose regulates ERK, JNK and p38 pathways to control neuronal survival and death remains to be further investigated. Additionally, we should emphasize that our findings were based on *in vitro* studies on rat adult NSCs. Although the OGD model partially mimics both ischemic and hypoxic insults, *in vivo* investigations remain to be conducted for a better understanding of the effect of glycemic control on adult NSCs.

## Competing interests

No authors declared any potential conflicts of interest.

## Authors’ contributions

Jian Chen made contributions to the study design, established the OGD model and drafted the manuscript. Yang Guo carried out western blot analysis, immunoassays, and statistical analysis. Wei Cheng participated in flow cytometry. Ruiqing Chen carried out the EdU incorporation assay. Tianzhu Liu participated in the western blots. Zhenzhou Chen contributed to the statistical analysis and critically revised the manuscript. Sheng Tan conceived the study, participated in its design and coordination, and helped to draft the manuscript. All authors read and approved the final manuscript.

## Supplementary Material

Additional file 1: Figure S1 The viability of NSCs was examined by MST assay after 24 h, 48 h or 72 h of growth. Neural stem cells were exposed to 2 mM, 7 mM or 17.5 mM D-glucose. The absorbance at 490 nm is directly proportional to the number of cells in each well at each time point. **Figure S2.** The relative increase in the number of neural progenitor cells in each group is represented by the ratio of 3-day viability to 1-day viability. The relative increase in NSCs exposed to 2 mM and 7 mM glucose was less than that in NSCs exposed to 17.5 mM glucose. The data are presented as the mean ± SD (n = 6) of the relative increase in cell number. *P < 0.05; NS, not significantly different. Click here for file
