# Supplementary material for: High glucose induces apoptosis and suppresses proliferation of adult rat neural stem cells following in vitro ischemia
Source: BMC Neurosci. 2013 Mar 4;14:24. doi: 10.1186/1471-2202-14-24 (PMC3599336; doi:10.1186/1471-2202-14-24)
Supplement: Additional file 1: Figure S1 — The viability of NSCs was examined by MST assay after 24 h, 48 h or 72 h of growth. Neural stem cells were exposed to 2 mM, 7 mM or 17.5 mM D-glucose. The absorbance at 490 nm is directly proportional to the number of cells in each well at each time point. Figure S2. The relative increase in the number of neural progenitor cells in each group is represented by the ratio of 3-day viability to 1-day viability. The relative increase in NSCs exposed to 2 mM and 7 mM glucose was less than that in NSCs exposed to 17.5 mM glucose. The data are presented as the mean ± SD (n = 6) of the relative increase in cell number. *P < 0.05; NS, not significantly different. [file 1471-2202-14-24-S1.doc]

**Supplemental information**

**High glucose induces apoptosis and suppresses proliferation of adult rat neural stem cells following *in vitro* ischemia**

**Jian Chen^1^*, Yang Guo^1^*, Wei Cheng^1^, Ruiqing Chen^1^, Tianzhu Liu^2^, Zhenzhou Chen^2^, Sheng Tan^1§^**

**Inventory of Supplemental Information**

Figure S1

Figure S2


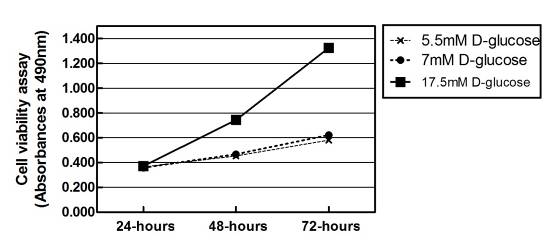


**Figure S1** The viability of NSCs was examined by MST assay after 24 h, 48 h or 72 h of growth. Neural stem cells were exposed to 2 mM, 7 mM or 17.5 mM D-glucose. The absorbance at 490 nm is directly proportional to the number of cells in each well at each time point.


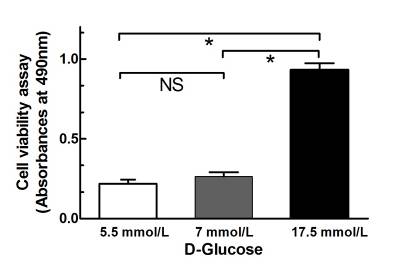


**Figure S2** The relative increase in the number of neural progenitor cells in each group is represented by the ratio of 3-day viability to 1-day viability. The relative increase in NSCs exposed to 2 mM and 7 mM glucose was less than that in NSCs exposed to 17.5 mM glucose. The data are presented as the mean ± SD (n = 6) of the relative increase in cell number. *P < 0.05; NS, not significantly different.
